# Qualitative Evaluation of Baduanjin (Traditional Chinese Qigong) on Health Promotion among an Elderly Community Population at Risk for Ischemic Stroke

**DOI:** 10.1155/2015/893215

**Published:** 2015-09-21

**Authors:** Guohua Zheng, Qianying Fang, Bai Chen, Hongmei Yi, Qiu Lin, Lidian Chen

**Affiliations:** ^1^College of Rehabilitation Medicine, Fujian University of Traditional Chinese Medicine, Fuzhou 350122, China; ^2^Department of Physical Education, Fujian University of Traditional Chinese Medicine, Fuzhou 350122, China; ^3^Fujian University of Traditional Chinese Medicine, Fuzhou 350122, China

## Abstract

*Background*. Baduanjin is a traditional Chinese qigong that has been practiced for a long time in China as a mind-body exercise in community elderly populations. The objective of this study was to qualitatively evaluate the perceived benefit of regular Baduanjin qigong in community elders. *Methods*. A total of 20 participants who had completed the 12-week Baduanjin qigong training were interviewed regarding their perceived effect on physical and psychological health and whether Baduanjin qigong was suitable for the elderly. *Results*. Almost all participants agreed that Baduanjin qigong could promote their multisystem or organ functions (e.g., digestive and circulatory systems), increase their immunity, make their bodies relax, and improve their mood and confidence. Most of the participants also agreed that Baduanjin qigong was appropriate for elderly individuals. Few individuals felt bored because of an hour Baduanjin training each day. *Conclusions*. The findings suggest that regular Baduanjin qigong may be potentially helpful to promote the overall physical and psychological health of elderly community populations and may be useful and feasible as a body-mind exercise in the health promotion in the elderly community populations.

## 1. Background

Baduanjin qigong has a long history in China as a comprehensive mind-body practice that involves physical postures and movement, breathing exercise, and relaxation, and it has been recommended for general use in the community by the Chinese Health Qigong Association [1, 2]. Its scientific and medical merits have received particular attention in recent decades. An increasing number of studies have investigated the sustained benefits of regular Baduanjin practice in preventing bone loss, reducing oxidative stress, and increasing antioxidant enzymes in middle-aged women, as well as increasing the quality of life and sleep quality in elderly community adults [3–5]. Additionally, effective improvement has also been demonstrated in balance and strength and in promoting physical flexibility in healthy adults [2, 6]. In recent years, more studies have focused on whether Baduanjin is effective in controlling the risk factors of cardiovascular and cerebrovascular diseases such as reducing blood pressure, insulin resistance, glycosylated haemoglobin, and blood glucose levels [7–12]. However, the positive effects of Baduanjin qigong have not been sufficiently confirmed by rigorous prospective trials.

Moreover, the majority of previous studies have only considered the variation between Baduanjin qigong and physical or psychological outcomes by quantitative measurement. Little information is available on the relationship between Baduanjin qigong and the practitioner's subjective experiences, which might present new outcomes. As a complex holistic “mind-body exercise” with the obvious characteristics of traditional Chinese medicine [13–15], Baduanjin qigong may be related to more health potential. Qualitative research, which collects data on the lived experiences of participants, is the perfect tool for exploring such complex experiences; it can not only help to improve our understanding of its benefits but also provide further knowledge about the phenomenon being studied [16]. In addition, such methods may detail experiences that are difficult to be captured through quantitative research and enrich our scientific knowledge beyond what can be gained quantitatively [17]. Therefore, based on a randomized controlled trial (RCT) with the aim of systematically evaluating the preventive effect of Baduanjin qigong on risk factors of ischemic stroke in an elderly community population (ChiCTR-TRC-13003588) [18], we performed a qualitative investigation to gain a deeper understanding of individual perspectives of participants after undergoing 12 weeks of Baduanjin training, to provide valuable supplementary information to evaluate the effect of Baduanjin qigong on an elderly community population.

## 2. Methods

### 2.1. Participants

Participants in the current qualitative study were recruited from those who had completed the Baduanjin qigong training program that took part in a RCT [18]. In the RCT, a total of 170 elders aged 50 to 70 years with risk factors of ischemic stroke were recruited from three communities (Wufeng, Fengdanbailu, and Chuntian) in Fuzhou city and randomly allocated into the Baduanjin qigong group and the usual physical activity group. The elders in the Baduanjin qigong group received a 12-week Baduanjin qigong training with a frequency of 40 minutes each time and 5 times per week. The training scheme of Baduanjin qigong complied with the “Health Qigong Baduanjin Standard” enacted by the State Sports General Administration in 2003 [19]. Two senior coaches who had engaged in teaching of Baduanjin qigong for at least five years were assigned to instruct their training. The coaches would interpret the inner connotations of each posture in detail before training until each elder could comprehend them. The elders in the usual physical activity group maintained their routine habit of physical activity. The quantitative measurements including cerebrovascular function, cardiopulmonary function, and physical parameters were measured at baseline and after the 12-week intervention duration.

Because of personal and time constraints, purposive samples of twenty participants (9 women, 11 men) with median age of 60 years and range from 50 to 69 years from Baduanjin qigong group were selected to participate in the semistructured interviews by using convenient sampling method. Seventeen of them had low levels of education (i.e., a high school education), and three participants had a college or higher level of education. Most of them had hypertension (15/20) or dyslipidemia (17/20); 13 participants were overweight. The participant characteristics were shown in Table 1.

### 2.2. Data Collection

Data were collected from August 1 to September 30, 2014, after the 12-week Baduanjin qigong training program. Two of the authors (Qianying Fang and Bai Chen) created an interview agenda mainly designed to explore subjective experiences of Baduanjin qigong for body-mind health, including experiences with Baduanjin qigong training, body and mind changes associated with Baduanjin qigong, and suitability of Baduanjin qigong for elders in community (Appendix). Each individual interview was conducted face to face by the authors (Qianying Fang and Bai Chen) at a quiet room of the participant's home. The interview scope gradually extended to focus on two main topics: (1) body and mind changes of the individual after the Baduanjin qigong training and (2) whether Baduanjin qigong is suitable for the elderly and why. The participants were encouraged to speak freely and to express their own views. Each interview was audio-recorded with participant permission and took at least 40 minutes. Additional data were also collected after each interview in the form of interviews' written notes and personal observations. The tape-recorded interviews were transcribed verbatim into Chinese, and the transcribed text was randomly double-checked.

### 2.3. Data Analysis

According to the qualitative content analysis, as described by Graneheim and Lundman (2004) [20], the data analysis was performed as follows. First, all transcribed words were read several times to obtain a sense of the entire meaning. Then, the participants' subjective experiences after the Baduanjin qigong training were extracted and combined. Third, the text was condensed and divided into different meaning units. The meaning units were further condensed and labelled with codes. Examples of the meaning units, condensed meaning units, and codes are shown in Table 2. Next, the various codes with similar content were sorted into categories. Then, the categories were sorted and formulated as subthemes. Finally, the subthemes were abstracted into themes. Examples of codes, subcategories, categories, and themes are provided in Table 3. The meaning units, categories, subthemes, and themes were checked and rechecked to ensure their stringent and trustworthy interpretations. All authors reflected on and discussed the codes, subthemes, and themes during the data analysis until a consensus was reached.

### 2.4. Ethical Considerations

The study was approved by the Medical Ethics Committee at the Affiliated People's Hospital, Fujian University of Traditional Chinese Medicine (approval number: 2013-021-02). The participants received both verbal and written information about the study and provided informed consent prior to study participation.

## 3. Results

The participants expressed their experiences on Baduanjin qigong in terms of “being sceptical,” “being suited for the elderly,” “harmonizing body and soul,” and “feeling bored.” The themes and subthemes are summarized in Table 4. The themes demonstrated an experiential process in which the participants' view changed after the Baduanjin qigong training. In the beginning, seven participants were sceptical whether Baduanjin qigong was effective and suitable for the elderly. However, as the Baduanjin qigong training scheme progressed, they gradually experienced the benefit of Baduanjin training for their mind-body health. All participants were surprised that Baduanjin qigong was easy to learn and had fewer environmental constraints. All participants agreed that Baduanjin qigong was suitable for elderly people to promote their whole health. Only one participant thought that Baduanjin training for 40 minutes each day was unacceptable and joyless. No participant experienced discomfort from the Baduanjin qigong. The themes are reported in the following headings with the subthemes in italics.

### 3.1. Being Sceptical at First

At the beginning of training, seven participants were sceptical about the efficacy or suitability of Baduanjin qigong in terms of health promotion. These participants talked about “doubting the efficacy” and “doubting the suitability” of Baduanjin qigong. However, with progression of the Baduanjin training program, they gradually experienced its benefit, which dispelled their doubts.

#### 3.1.1. Doubting the Efficacy

Three participants doubted whether the Baduanjin qigong could truly benefit their physical and mental health because they thought that the exercise intensity of Baduanjin might be lower than that of other physical exercises, such as jogging, brisk walking, and square dancing. The effects of Baduanjin qigong on health promotion in elderly adults were slight; there was no effect in some cases. One participant had previously learned Baduanjin qigong by watching a TV program and practiced it intermittently for a longer period, but this participant did not experience any obvious health benefit.

*Is it really effective for my health? (ID 12, male, 67 years old, with dyslipidemia, hypertension and overweight)*

*It is composed of eight movements and appears to be simpler than other exercises. (ID 3, male, 60 years old, with hypertension)*

*I know that Baduanjin qigong is good for my health, I learned it from TV, but I did not experience any benefit. (ID 9, male, 61 years old, with dyslipidemia)*

#### 3.1.2. Doubting the Suitability

Four participants did not have the confidence to learn Baduanjin qigong because of their bad memories and learning abilities. Most of them felt that it would take a long time to grasp the connotation of Baduanjin qigong, although it only consisted of eight postures.

*Maybe I cannot grasp it. (ID 19, male, 62 years old, with dyslipidemia, overweight, smoker and a family history of stroke)*

*I don't think it's suitable for me even though Baduanjin has only eight postures. I can never remember things because of my bad memory. (ID 16, female, 57 years old, with dyslipidemia, overweight)*

### 3.2. Being Suited for the Elderly

Most of the participants found that Baduanjin qigong was suitable for elderly people after the Baduanjin qigong training protocol was completed. They said that Baduanjin qigong was a “moderate amount of exercise,” was “simple and easy to learn,” and had “fewer environmental constraints.”

#### 3.2.1. Moderate Amount of Exercise

Seventeen participants said that the level of usual exercises, such as square dancing, swimming, and softball, was too high for elderly people to adhere to practicing for 45–60 minutes each day; in addition, the movements of Baduanjin qigong were gentle and slow and provided low-moderate intensity exercise. Most of participants were able to practice Baduanjin qigong for one hour each day.

*In the past, I square-danced, I found that it was difficult for me to continue, maybe it was a problem because of my weight. I can't catch the rhythm of dance music. I felt uncomfortable and tired. I hated my fat body…However, now, I am so happy to exercise. The most mysterious part of Baduanjin qigong is that you soon get warm, but you may never get tired. (ID 2, female, 50 years old, with hypertension, overweight and family history of stroke)*

*Five years ago, swimming was my favourite sport. After I got stomach cancer, I often felt weak, and swimming no longer fit me. The level of Baduanjin qigong is lower than swimming, it provides a moderate amount of exercise, and I can practice Baduanjin qigong every day. (ID 5, male, 64 years old, with hypertension, diabetes mellitus and overweight)*

*There was no way to exercise with my waist pain, but I can perform Baduanjin qigong. It eased my pain. It is a gentle movement. (ID 14, female, 57 years old, with hypertension, dyslipidemia, overweight and a family history of stroke)*

#### 3.2.2. Simple and Easy to Learn

All participants were surprised that it was easy for elderly adults to learn Baduanjin qigong training. They said that Baduanjin qigong was only composed of eight postures involving movement of the arms, legs, and waist and that it was simple and did not require many skills.

*It only has eight movements! It is easy for us to accept and learn. You know, our memory is poor, it's difficult for us to learn a new exercise, but Baduanjin qigong is simple, slow, and relaxing. It only took me one week to master it. (ID 13, female, 65 years old, with hypertension and dyslipidemia; uses amlodipine tablets)*

#### 3.2.3. Less Environmental Constraint

Twelve participants reported that they could practice Baduanjin qigong at home at any time because a large training space or other auxiliary apparatuses were unnecessary. They said that they could not find, thus far, another exercise that was more convenient than Baduanjin qigong.

*I like to exercise, but it is always trouble for me. A sunny day and an exercise space area are necessary to practice, but it is difficult to get both at the same time. Now all problems are solved, I can perform Baduanjin qigong on my balcony without any equipment once I am free. (ID 17, female, 60 years old, with hypertension, dyslipidemia and overweight)*

*Today is a busy day for my kids, I will perform Baduanjin qigong at home. I do it at home on rainy days. I think Baduanjin qigong is convenient, and only two things are needed: a place to stand and background music for the Baduanjin qigong. (ID 14, female, 57 years old, with hypertension, dyslipidemia, overweight and a family history of stroke)*

*At first, I practice this exercise following the background music and a coach. Now I can do it at home without the music; the background music seems to be played in my mind. (ID 7, male, 63 years old, with dyslipidemia, overweight and a family history of stroke)*

### 3.3. Harmonizing Body and Soul

All participants described the experiential process of Baduanjin qigong harmonizing their body and mind. They talked about how Baduanjin qigong “improved physiological health” and “improved psychoemotional state” after the training.

#### 3.3.1. Improving Physiological Health

With the progression of Baduanjin qigong training, all participants became more aware of their bodies and how their bodies felt. They perceived improvements in their bodies and changed in various ways, including “relieving pain,” “enhancing digestion,” “relieving polyuria,” “strengthening immunity,” “improving the function of circulatory system,” and “improving sleep quality.”

*Relieving Pain*. Five participants found that Baduanjin qigong could relieve pain. After they had practiced Baduanjin qigong for a few days, they found their muscular soreness, headache, or low back pain relieved gradually and even vanished later. Four participants described that they could feel the effect of* qi* (i.e., a vital energy within the body according to traditional Chinese medicine theory) after Baduanjin qigong training for several weeks.

*The coach told us to adjust breathing to make the process of breathing smooth, then felt qi went up from the Dantian (i.e. a focal point for qigong exercise technique as well as the centre of balance and gravity). At first, I couldn't understand, but after a few days, I really felt it. It was unbelievable; I felt that the qi went up to my chest, my nose and my head, then my stuffy nose gradually improved, and my headache was disappeared. (ID 2, female, 50 years old, with hypertension, overweight and a family history of stroke)*

*Baduanjin qigong is very good at helping me to exercise. Because of taking care of my grandchildren, I had suffered from low back pain for quite a long time. My low back pain has been alleviated more and more after practicing Baduanjin qigong. It has not occurred again. It's really benefit to me. (ID 13, female, 65 years old, with hypertension and dyslipidemia; uses amlodipine tablets)*

*I had a headache and back pain before, but now I do not. When my headache or back pain attacked previously, I always used massager, but now the massager is fired. (ID 11, female, 69 years old, with hypertension, dyslipidemia, overweight, diabetes mellitus and a family history of stroke; uses amlodipine tablets)*

*When I came here to participate in the Baduanjin qigong training, I could not raise and stretch my arms because of shoulder periarthritis. However, now look me, I can raise my arm easily without any pain. It is marvellous. (ID 8, male, 69 years old, with hypertension, diabetes mellitus and smoking; uses amlodipine, metformin, and acarbose tablets)*

*Enhancing Digestion*. Eight participants reported that their digestion gradually improved. They described having better appetites after participating in the Baduanjin qigong training and that these changes gradually developed, even if they were not aware of the changes taking place. Six participants also reported that their flatus and distension issues disappeared after they practiced Baduanjin qigong. Three participants had suffered from intractable constipation or flatulence for several years. Now, after practicing Baduanjin qigong for several weeks, they have regular defecation without constipation or flatulence.

*I benefit from Baduanjin qigong; otherwise I would not have participated for so long time. Recently my daughter told me that I eat more than I have in the past. At that time, I realized that my good appetite had returned. I used to not eat dinner because had no appetite. Now I have a good appetite. (ID 13, female, 65 years old, with hypertension and dyslipidemia; uses amlodipine tablets)*

*I had intractable constipation for several years. Currently, I have a bowel movement every morning after breakfast. I don't need to sit on the night stool in agony for hours. I no longer endure constipation for several days; now I have more regular bowel movements than before. (ID 16, female, 57 years old, with dyslipidemia, overweight)*

*I used to have uncomfortable flatulence after meal. The foods always made my stomach bloated, so I only ate a little. However, it's much better now. When I do the first or third posture of Baduanjin qigong, in which two hands reach up to the heavens to regulate San Jiao, and raise my hands on each side to adjust the spleen and stomach, I have the hiccups or fart. I feel very comfortable. (ID 10, male, 69 years old, with hypertension, dyslipidemia and smoking; uses benazepril tablets)*

*Relieving Polyuria*. Two participants with polyuria reported that the frequency of their polyuria or nocturia was obviously decreased after they practiced Baduanjin qigong for several weeks. Both participants were surprised at the efficacy of Baduanjin qigong on their polyuria.

*I went to the toilet approximately once an hour, but the volume of urine was small every time. However, I could not tolerate it if I didn't go to the toilet. After I practiced Baduanjin qigong each day for one month, the frequency of urinating decreased. Now I have control of the situation, and I have few nocturnal urination. (ID 1, female, 58 years old, with hypertension, dyslipidemia and overweight)*

*Strengthening Immunity*. Two participants reported their experiences related to immunity. One female participant said that her friends were surprised at her good complexion, but the best result was that she had more physical strength than ever, and now she could perform some housework. A participant who smoked felt that his ability to resist disease was much better after performing Baduanjin qigong for several weeks.

*One day, my friend stared at me in amazement. She told me that I look so good after the Baduanjin qigong. My feeling is that I am not easily fatigued, it seems that I have more physical strength. …I can travel with my family and go hiking with my friends. (ID 6, female, 61 years old, with hypertension, dyslipidemia, diabetes mellitus and overweight, uses amlodipine, metformin tablets)*

*I have smoked for a long time, and I used to be susceptible to catching colds, but now I feel much better. Now I practice Baduanjin qigong every day, and I seem to get more strength. (ID 15, male, 66 years old, with hypertension, dyslipidemia, overweight and smoking; uses nitrendipine tablets)*

*Improving the Circulatory System Function*. Three participants reported that their blood pressure had gradually decreased after Baduanjin qigong. One participant with hypertension described the changes to his blood pressure. He has taken hypotensive drugs for 10 years. After training in Baduanjin qigong for two months, he found that his blood pressure had returned to normal.

*I have hypertension. I previously took hypotensive drugs every day. However, my blood pressure was gradually lowered when I performed Baduanjin qigong for two months. Now my blood pressure is almost normal, and I have stopped taking the hypotensive drugs. …I measure my blood pressure every day. In the past, my blood pressure had large fluctuations within one day. The systolic blood pressure was approximately 150 mmHg, 130 mmHg and 150 mmHg in the morning, noon and night. Recently, my systolic blood pressure stabilized to 130 mmHg, 125 mmHg and 130 mmHg in the morning, noon and night. It's more normal and stable than before. (ID 10, male, 69 years old, with hypertension, dyslipidemia and smoking; uses benazepril tablets)*

*Improving Sleep Quality*. Seven participants described that the quality of their sleep became better during practicing Baduanjin qigong. It was difficult for them to fall asleep before; furthermore, they experienced insomnia and dreamful sleep and were easily awoken. After they began practicing Baduanjin qigong, they noticed that their sleep had improved, their sleep time was extended, and they fell asleep quickly.

*In the past, although I lay down on my bed with eyes closed, the messy thinking in my brain was still working. You know, the more I want to sleep quickly, the more I cannot sleep. I was scared to sleep. …After a few weeks of training Baduanjin qigong, I can fall asleep as soon as I lied down. It's unbelievable! I never had such a good sleep quality. (ID 2, female, 50 years old, with hypertension, overweight and a family history of stroke)*

#### 3.3.2. Improving Psychoemotional State

All participants reported that the changes in their mind-body connectedness resulted in a sense of wholeness. Sixteen participants felt comfortable and relaxed after the Baduanjin qigong. One participant shared her artistic conception of Baduanjin qigong training: she imaged herself as a tree in the nature when she was practicing Baduanjin qigong; then she could soon feel relaxed and understand the key purpose of the movements. Eight participants reported that Baduanjin qigong made them calm, pleasurable, and anxiety-free. They said that they enjoyed their exercise and seemed to obtain more vigour from it. Four participants also said that they obtained positive psychological energy and felt full hopeful about their lives.

*When I raise one hand while pressing the other hand downward (the second posture of Baduanjin qigong), my abdominal cavity was extended along with this movement. I felt comfortable. (ID 1, female, 58 years old, with hypertension, dyslipidemia and overweight)*

*The coach told us try to melt our body into the nature. When I stood here to practice Baduanjin qigong, I image myself as a tree. I breathed slowly along with the movement spreading slowly. …It makes me relax quickly. I think it would help me in a relaxed nature sense. My body needs it. (ID 13, female, 65 years old, with hypertension and dyslipidemia; uses amlodipine tablets)*

*I'm in a good mood after Baduanjin qigong. My daughter always complained she had a dad with bad temper. When I met someone I didn't like, I would angry with him or her. Now I become more clam and patient, and my family relationship is more harmonious. That's all because of Baduanjin qigong. (ID 10, male, 69 years old, with hypertension, dyslipidemia, and smoking; uses benazepril tablets)*

*It's a positive energy! All is developing toward a good direction. I'm a post-operative gastric cancer patient, but now I feel hopeful about my life. My blood pressure is stable, and my hands feel more powerful. (ID 5, male, 64 years old, with hypertension, diabetes mellitus and overweight)*

### 3.4. Feeling Bored

One participant described that she felt that Baduanjin qigong was boring and monotonous because of its eight simple movements. She said it was difficult to maintain Baduanjin qigong training for 40 minutes every day for 12 weeks.

*It seems boring and monotonous if I perform Baduanjin qigong for 40 minutes, I cannot concentrate my attention on one thing. (ID 18, female, 50 years old, with hypertension, dyslipidemia and atrial fibrillation)*

## 4. Discussion

This qualitative investigation first explored the experience and subjective feelings of elderly community adults for 12 weeks of Baduanjin qigong training. Four likely interconnected themes emerged from data analysis. The first theme: the effect of Baduanjin qigong is “being sceptical at first.” In the regimen of traditional Chinese medicine (TCM), qigong is a form of “mind-body” exercises, which simultaneously harmonize the “mind” and the “body.” “*Qi*” in TCM refers to vital energy within the body. Free flow of “*qi*” within the meridian system is essential for good health, whereas illness would occur if* qi* was blocked. “Gong” means training and practice.* Qigong* can promote the healthy life through strengthening or balancing free flow of internal* qi* during consistent practice [21]. Current evidences have demonstrated that qigong has positive effects on physical and psychological health [22, 23]. Baduanjin (also translated as eight pieces of brocade, eight silken movements, or eight silk weaving movements in English) is a complete set of qigong exercises based on TCM theory, containing meditation, breathing, body posture, and gentle movement. It has been practiced for more than one thousand years as a typical exercise to promote health in China [1]. In practicing, the mind, body, and breath are required to be smooth and unstrained. Only together breathing, body, and mind are trained to work in harmony by practicing Baduanjin, practitioner can obtain positive effect of Baduanjin qigong [24]. However, this effect is difficult to be achieved in case of failure to be correctly practiced. In this study, some participants doubted the effect of Baduanjin qigong for their health at the beginning of training. It is reasonable for them because they did not understand and master the training process of Baduanjin qigong at beginning of training. Similarly, for the fourth theme (“feeling bored”), it also is possible to feel bored and monotonous if practitioner does not correctly master the inner essence of Baduanjin qigong at practicing. These findings suggest that it is key how to harmonize breathing, posture, and mind when practicing Baduanjin qigong. It also implies that it needs more guidance and explanation on the inner connotation of Baduanjin to practitioners in the future study.

The second theme: Baduanjin qigong can harmonize practitioner's body and soul (“harmonizing body and soul”). Baduanjin improved physical function and capacity, being reasonable in terms of the physiological effect of Baduanjin qigong. Though Baduanjin is only composed of eight separate postures (i.e.,* Liangshou tuo tian li sanjiao, Zuoyou kai gong si she diao, Tiaoli piwei xu dan ju, Wulao qishang xiang houqiao, Yaotou baiwei qu xinhuo, Shangshou pan zu gu shen yao, Zan quan numu zeng liqi, and Beihou qidian baibing xiao*), each of them focuses on a different physical area (particular organ or system) and meridian which is a traditional Chinese medicine belief about a path through* qi* flows [25]. It satisfies its goal of balancing* yin* and* yang* by regulating* qi*, strengthening tendons, and improving bone strength. For instance, the third posture of Baduanjin qigong, which is called “*Tiaoli piwei xu dan ju*,” helps regulate the spleen and stomach through moving hands up and down over the head. The spleen (*pi*) and stomach (*wei*) are vital in the digestion and absorption processes, which are a main energy source of the human body, according to traditional Chinese medicine (TCM) [26]. The sixth posture is called “*Shangshou pan zu gu shen yao*” in which the practitioner uses both hands to grasp the toes along the legs. This motion can strengthen the kidneys and the waist and benefit to increase function of urinary and immune systems [27]. Therefore, when Baduanjin is practiced, the practitioner's body maintains a steady centre of gravity that drives the movement of the four limbs using the lumbar spine as the axis. Meanwhile, the muscle tension and relaxation alternate at different parts of the body, and the breathing is smooth and unstrained [28, 29]. With regular practice and rehearsal of the gentle movement as well as the atonement of breath, practitioners may achieve greater improvement in strength, physical fitness, and function of multiorgans and systems. A recent quantitative RCT also reported that the 16-week Baduanjin training could significantly improve some physical fitness indices including physical flexibility, subcutaneous adipose accumulation, and muscles strength in healthy adults, but the significant variations were not detected in blood pressure and cardiorespiratory capacity indices [30]. With regard to psychology and mind, Baduanjin qigong could make practitioners' mind relaxed, calm, and pleased and therefore is beneficial to improve the psychological and mental health. Different from other aerobic exercises, Baduanjin can coordinate the mind and body by controlling the flow of* qi* in the body, which is called vital energy to maintain the healthy body [27]. From the perspective of Chinese medicine, the circulation of* qi* is closely related to psychological and mental health. The negative psychology such as angry, depressive, irritable, and restless mood may cause the stagnation of* qi*. On the other hand, the normal circulation of* qi* is also helpful to regulate the passive emotions [31]. Baduanjin qigong focuses on the mobilization of functional potentialities, makes the process of breathing smoother, and unifies the mind and body by regulating breathing, thereby promoting the normal circulation of* qi*. In this interview, participants felt their body relax, mood calm, even better their bad temper after regular Baduanjin training for several weeks. The findings indicate Baduanjin qigong's potential to improve practitioners' physical function and its benefit to promote their psychological and mental health. Consistent with the current study's findings, many quantitative studies found that regular practicing of Baduanjin qigong could promote the physical and mental health in the general population and for those with specific health challenges. For example, practicing Baduanjin could increase the capacity for physical activity and physical fitness in adults [32, 33], enhance cardiopulmonary function [34], relieve anxiety and stress [35, 36], and improve sleep quality [37].

The third theme: Baduanjin qigong is suitable for elderly populations (“being suited for the elderly”). Baduanjin is one of the more common forms of qigong, which is a relatively simple set of exercises (only eight movements) that can be completed in 20 min or less [29]. Previous studies have suggested that Baduanjin had few physical and cognitive demands and was easy to practice [9]. Furthermore Baduanjin provides low-moderate exercise intensity [5]. All participants in the current study reported that Baduanjin qigong was simple and easy to learn and had no environment constraints. It therefore is suitable for elderly populations.

This study has several strengths and potential limitations. A strength of the current study was that this qualitative investigation was based on a randomized controlled trial (RCT) evaluating the complex efficacy of Baduanjin qigong intervention for which the protocol of this RCT has been published in* Trials* [38]. Current qualitative study should help to enhance the evidence of effectiveness produced by the RCT or facilitate the feasibility of the RCT itself [39]. A possible limitation of this study was that all interviewees were drawn from a community RCT; representatives from that trial population are likely to differ in some respects from the whole population. Thus, it is possible that the generalization of these study results is limited. In addition, this study used the purposive sampling only from the Baduanjin qigong group rather than random sampling method to select samples from all participants, and the present sample size represented a relatively small proportion (*n* = 20) of the total RCT participants (*n* = 170). A potential selective bias may have resulted from the selection of samples for this qualitative study. However, all participants were encouraged to talk in depth about their perspectives of the research topic. Furthermore, when we found parts of interviews that required modification, we visited the same participants for a second time.

## 5. Conclusions

The results of present study suggest that the practice of regular Baduanjin qigong training provides a number of benefits that promote the body and mind health among community elderly population. Participants reported a wide range of benefits, including promoted digestive function, circulatory system function, immunity and sleep quality, relaxed body and moods, and enhanced self-tranquility, pleasure, and symptom relief. Meanwhile they also thought that Baduanjin qigong was a suitable exercise for elderly community adults. These results indicate that Baduanjin qigong is a potentially helpful and feasible mind-body exercise that promotes the body-mind health of elderly community populations.

## Figures and Tables

**Table 1:** Participants' characteristics.

Characteristics	Intervention group *n* = 20
Female	9
Male	11

Age (range year)^1^	60 (50–69)

Marital status	
Married	20

Education	
Elementary school or below	5
Junior high school	4
Senior high school	8
College and above	3

Risk factors of ischemic stroke	
Hypertension	15
Atrial fibrillation	1
Smoking	3
Dyslipidemia	17
Diabetes mellitus	4
Overweight	13

^1^Median (range).

**Table 2:** An example of meaning units, condensed meaning units, and codes.

Meaning unit	Condensed meaning unit	Code
I had suffered from low back pain for quite a long time. The low back pain has been alleviated more and more after exercising Baduanjin.	The low back pain has been alleviated more and more	The low back pain has been alleviated

My right arm always put me in an unbearable suffering; I could not raise it. Now, you look at me; I can raise my arm easily without pain.	The right arm is without pain and can be raised easy	Arm without pain

**Table 3:** An example of codes, subcategories, categories, and a theme from qualitative content analysis about the experiences of practicing Baduanjin.

Theme	Harmonizing body and soul						
Category	Improving physiological health						Improving psychoemotional state

Subcategory	Relieving pain	Enhancing digestion	Relieving polyuria	Strengthening immunity	Improving the circulatory system function	Improving sleep quality	

Codes	The low back pain has been alleviated; arm is raised without pain; headache and back pain seem to be disappeared	Having a good appetite; bowel movement becomes regular; constipation is decreased	Reducing frequency of urination and nocturnal urination	Looking fine; being seldom sick	Controlling blood pressure; relieving dizziness	Being fast asleep; extending sleep time	Having positive vital energy; whole body is relaxed; being comfortable; being hopeful; being full of vital energy; being not lonely; being anxiety-free

**Table 4:** Overview of subthemes and themes.

Subtheme	Theme
Doubting the efficacy	Being sceptical
Doubting the suitability	

Moderate amount of exercise	Being suited for the elderly
Simple and easy to learn	
Less environmental constraint	

Improving physiological health	Harmonizing body and soul
Improving psychoemotional status	

Boring and monotonous	Feeling bored

## Appendix

### The Outlines of Interview

Date of interview: …
Interviewer: …
Subject initials: …

*Introduction*

- Thank you for coming in for this interview.
- Purpose: To explore how you think about Baduanjin exercise. Hear your experiences of the program. Learn more about your experience that we may have missed on the physical measurements.
- Confidentiality: Your answers will never be linked to your name. You will remain anonymous. Only study staff will have access to the recordings and transcripts.
- These interviews usually take about 40 minutes.
- If there are any questions that you do not want to answer. Please let me know.
- Do you mind if we tape the interview? Yes/No (turn on recorder).

*(1) Experience-Narrative of Baduanjin Exercise*

- Please tell me how Baduanjin exercise program were for you? (if no spontaneous narrative: Please tell me any stories from the exercise program.)
- Can you tell me how you feel after the Baduanjin exercise?
- What do you think about Baduanjin exercise?

*(2) Have You See Any Changes in Your Body or Mind because of the Baduanjin Exercise?*

- Have you noticed any changes in your health?
- Can you narrate the changes in detail?
- Do you think these changes in your health result from Baduanjin exercise?
- How about your sleep and mood?
- How do you think Baduanjin exercise work?

*(3) Is Baduanjin Exercise Suitable for Elderly?*

- Do you feel the difficulty to learn the postures of Baduanjin exercise?
- Can you understand the relationship the postures of Baduanjin exercise and your breath?
- Have you used any of the tools when you learned or practiced Baduanjin exercise?
- Do you feel tired after completed the Baduanjin exercise each day?
- Can you continue to practice Baduanjin exercise in your future life?

*(4) Closure*

- Do you have any questions about Baduanjin exercise program?
- How was this interview for you?

*(5) Observational Notes (Interviewer: Please Record Any Body Language, Other Cues)*
